# Virtual reality-enhanced collaborative learning and self-reported metacognitive awareness in a Chinese EFL context: the mediating role of social interaction and the moderating role of learner engagement

**DOI:** 10.3389/fpsyg.2026.1852175

**Published:** 2026-07-15

**Authors:** Ying Zhang, Feifei Wang, Hong Zhou

**Affiliations:** 1 Department of Special Education, Shandong Vocational College of Special Education, Jinan, China; 2 School of Humanities, Arts and Education, Shandong Xiehe University, Jinan, China; 3 Shandong University of Art & Design, Jinan, China

**Keywords:** EFL, learner engagement, self-reported metacognitive awareness, social interaction, structural equation modeling, virtual reality

## Abstract

This study examined the association between virtual reality (VR)-enhanced collaborative learning environments and self-reported metacognitive awareness among 520 Chinese university EFL students. Drawing on sociocultural theory and technology-enhanced learning frameworks, we proposed and tested a moderated mediation model in which social interaction mediates the VR–metacognition association, and learner engagement moderates the mediation pathway. Structural equation modeling (CB-SEM) with latent moderated structural equations (LMS) and bootstrap confidence intervals (5,000 resamples) was employed. Results indicated that VR-enhanced collaborative learning was positively associated with self-reported metacognitive awareness (*β* = 0.312, *p* < 0.001) and with social interaction (*β* = 0.441, *p* < 0.001). Social interaction was further associated with self-reported metacognitive awareness (*β* = 0.298, *p* < 0.001), conforming the indirect association (0.131, 95% CI [0.078, 0.192]). Learner engagement significantly moderated the social interaction–metacognition pathway (*β* = 0.178, *p* < 0.001), such that the indirect association was stronger among more highly engaged learners. These findings extend existing scholarship on technology-enhanced language learning and offer context-specific implications for designing VR-based collaborative learning in Chinese university EFL settings; their generalizability to other populations and instructional contexts remains to be established. Because the design was cross-sectional, the relationships reported are associational and should not be interpreted causally.

## 1 Introduction

The integration of emerging technologies into language education has generated sustained scholarly inquiry into the cognitive and social mechanisms through which digital learning environments may support language acquisition and broader academic competencies (Lee et al., 2025; Zhang and Hyland, 2025). Among these technologies, virtual reality (VR) has attracted growing attention as a medium capable of providing immersive, embodied, and socially rich experiences that may be associated with self-reported metacognitive awareness in ways that differ from conventional instruction (Roe et al., 2025; Watts, 2025). Despite proliferating enthusiasm for VR-based pedagogy, empirical investigations into the specific psychological pathways linking VR use to metacognition remain sparse, particularly within EFL contexts in which metacognitive regulation is closely tied to second language (L2) writing achievement and oral communicative competence.

Metacognition, encompassing learners’ capacities for planning, monitoring, and evaluating their own cognitive processes, has long been recognized as a foundational correlate of academic success (Abdelaty et al., 2025; Ranalli, 2018). Prior research has documented robust associations between self-reported metacognitive awareness and learner autonomy (Almelhes, 2025), between metacognitive monitoring and self-regulated writing performance (Abdelaty et al., 2025; Zainuddin et al., 2026), and between metacognitive instruction and improvements in L2 accuracy (Barrot, 2023). However, the mechanisms through which technology-enhanced collaborative learning environments relate to self-reported metacognitive awareness remain underspecified.

Social interaction has been theorized as a critical mediating mechanism linking technology use to cognitive outcomes (Li and Zhang, 2022; Peng et al., 2023). Within sociocultural frameworks, higher-order cognitive processes, including metacognitive regulation, are internalized through collaborative dialogue, negotiated meaning, and scaffolded interaction (Mujtaba et al., 2021; Sang and Zou, 2023). VR environments, by virtue of their immersive and embodied character, may potentiate socially mediated cognitive development in ways that exceed those achievable in conventional digital learning environments (Roe et al., 2025). Whether social interaction transmits VR affordances to metacognitive outcomes, and under what learner conditions this mediation is strongest, remains an open empirical question.

Learner engagement has emerged in the technology-enhanced learning literature as a boundary condition shaping the effectiveness of instructional interventions (Koltovskaia, 2020; Wang et al., 2025). Behavioral, cognitive, and emotional engagement have each been associated with more productive use of technology affordances. Whether learner engagement moderates the social interaction–metacognition pathway in VR environments constitutes a theoretically motivated research question.

The present study addresses these gaps by testing a moderated mediation model in which (1) social interaction mediates the VR–metacognition association, and (2) learner engagement moderates the social interaction–metacognition link. Data were collected from 520 Chinese university EFL students enrolled in VR-enhanced collaborative writing courses. The study contributes to expanding scholarship on technology-enhanced language learning (Lee et al., 2025; Lee, 2022) and on metacognitive self-regulation in technology-mediated contexts (Abdelaty et al., 2025; Zainuddin et al., 2026).

## 2 Literature review

### 2.1 VR-enhanced collaborative learning in EFL contexts

Virtual reality has increasingly been positioned as a transformative medium for language learning, owing to its capacity for immersive simulation of authentic communicative contexts (Roe et al., 2025). Roe et al. (2025) found that synthetic avatars deployed in immersive virtual environments could generate engagement and learning outcomes comparable to face-to-face instruction, underscoring the pedagogical potential of VR-mediated interaction. The experiential learning paradigm has also been applied to understand how technology-enhanced environments scaffold learners’ knowledge construction processes (Watts, 2025).

Recent systematic reviews converge on a common conclusion while leaving a key gap unaddressed. Across the collaborative-VR literature, shared immersive environments are repeatedly found to support authentic peer interaction and joint knowledge construction (Van der Meer et al., 2023); yet this benefit is conditional rather than automatic, materializing only when tasks are designed for genuine interdependence, shared-workspace use, and synchronous exchange (Paulsen et al., 2024). Where these conditions are met, VR has been associated with tangible language-learning gains—vocabulary acquisition and motivation among EFL learners (Chen et al., 2021)—and, more pertinent here, with opportunities for metacognitive planning and authentic communicative practice that are difficult to reproduce in conventional instruction (Parmaxi, 2023). What this work establishes is that the collaborative affordances of VR are real but design-contingent; what it has not yet specified is the mechanism through which those affordances reach metacognitive outcomes—the question the present study addresses.

Despite these advances, existing research has not yet systematically examined VR as a collaborative learning medium specifically targeted at metacognitive outcomes. The present study addresses this gap by situating VR within the broader ecology of technology-enhanced L2 learning environments and by specifying the social interactional mechanisms through which VR may relate to self-reported metacognitive awareness.

### 2.2 Social interaction as a mediator

Sociocultural theory posits that higher psychological functions, including metacognitive regulation, emerge through social interaction before becoming internalized as individual competence (Li and Zhang, 2022; Mujtaba et al., 2021). Empirical support for this claim has accumulated from research on collaborative feedback processing in L2 writing. Peng et al. (2023) reported that collaborative processing of teacher feedback was associated with deeper engagement and greater internalization than individual processing, suggesting that social interaction transforms externally provided feedback into internal regulatory resources. Similarly, Mujtaba et al. (2021) found that collaborative written corrective feedback processing was associated with greater accuracy in L2 writing, with social discussion serving as a mechanism through which metacognitive rules about language form were negotiated and consolidated.

Social presence, the perceived sense of being with others in a shared virtual environment, has emerged as a theoretically critical dimension of VR-mediated social interaction. Dunmoye et al. (2024) examined the association between social presence and cognitive engagement in collaborative VR learning environments, finding that higher perceptions of co-presence were associated with greater cognitive engagement, particularly on tasks requiring joint knowledge construction and collaborative problem-solving. Teng (2024) reported that VR digital gaming environments were associated with metacognitive autonomy through social interaction and community-building processes, underscoring the reciprocal relationship between social presence, learner engagement, and self-reported metacognitive awareness. These findings collectively suggest that the qualitative character of social presence, rather than the mere frequency of social exchange, may constitute the proximal mechanism through which VR-enhanced collaborative environments translate their technological affordances into cognitive and metacognitive gains.

### 2.3 Self-reported metacognitive awareness in technology-mediated learning

Metacognition encompasses learners’ awareness, monitoring, and regulation of their own cognitive activities, and has been identified as a key correlate of L2 writing proficiency and academic achievement (Abdelaty et al., 2025; Ranalli, 2018). Research on technology-enhanced learning environments has increasingly attended to how these environments may support metacognitive awareness. Abdelaty et al. (2025) found that self-regulatory processes, closely related to metacognitive monitoring, significantly mediated the relationship between feedback quality perceptions and L2 writing achievement. Almelhes (2025) proposed that learner autonomy, construed as a dimension of metacognitive self-direction, is constituted through intersecting dynamics of power, control, and metacognitive awareness in language learning environments.

The role of technology in supporting metacognitive awareness has been explored across digital learning tools. Zainuddin et al. (2026) found that e-portfolios as authentic assessment tools were associated with enhanced metacognitive awareness and self-regulated learning among pre-service teachers, highlighting the importance of reflective documentation in metacognitive awareness. Reynolds et al. (2021) reported that perceived source of feedback was related to L2 writing performance through metacognitive pathways, as learners’ beliefs about feedback credibility shaped their metacognitive engagement with revision.

A small but growing body of work has begun to trace how VR relates to metacognition, and two regularities emerge. First, the metacognitive yield of VR appears to depend on explicit structuring: embedding cognitive and metacognitive strategy prompts raises metacognitive regulation and inquiry performance, and does so more strongly in collaborative than in individual conditions (Chen, 2025), while rendering learners’ strategic patterns visible through analytics dashboards similarly supports self-reported metacognitive awareness (Geng and Yamada, 2025). Second, the reflective and social character of immersive environments matters: VR-mediated reflection has elicited more sophisticated metacognitive knowledge than text-based approaches (Rivers et al., 2021), an effect that extends beyond language learning to domains such as anatomy education (Neyem et al., 2026). Taken together, these findings point to social structuring—rather than immersion per se—as the active ingredient, foreshadowing the mediating role of social interaction examined here.

### 2.4 Learner engagement as a moderator

Learner engagement, broadly defined as the behavioral, cognitive, and affective investment of learners in educational activities, has been identified as a boundary condition for the effectiveness of technology-enhanced learning interventions (Koltovskaia, 2020; Wang et al., 2025). The broader premise that engagement is not merely an outcome but a condition shaping whether technological affordances translate into learning gains is well established across technology-enhanced learning research (Zhang and Hyland, 2018). Within immersive environments specifically, however, engagement acquires distinctive significance, because the social and embodied affordances of VR can be exploited only by learners who are actively invested in the collaborative task.

Evidence from VR and immersive learning contexts suggests that engagement governs how fully learners draw on the social affordances of shared virtual environments. Dunmoye et al. (2024) found that higher cognitive engagement in collaborative VR environments was associated with greater benefit from social presence, indicating that engagement conditions the link between co-presence and cognitive processing. Teng (2024) similarly reported that learners more deeply engaged in VR gaming environments derived stronger metacognitive autonomy from social interaction and community participation. These findings position engagement as a moderator that determines whether socially mediated interaction in VR is converted into metacognitive gains, rather than as a mere antecedent of those gains.

The moderating role of engagement in VR contexts is theoretically grounded in the concept of presence—the sense of being immersed in a virtual environment—which is understood to heighten behavioral and cognitive engagement (Roe et al., 2025). More highly engaged learners are expected to exploit VR’s social interactional affordances more fully, generating richer collaborative dialogue and more readily internalizing the metacognitive insights that emerge through social negotiation, thereby strengthening the pathway from social interaction to self-reported metacognitive awareness. This moderated mediation hypothesis constitutes the novel theoretical contribution of the present study.

### 2.5 Hypotheses development

On the basis of the foregoing review, we advance the following hypotheses:

*H1*: VR-enhanced collaborative learning is positively associated with self-reported metacognitive awareness (*β* > 0).

*H2*: VR-enhanced collaborative learning is positively associated with social interaction quality (β > 0).

*H3*: Social interaction quality is positively associated with self-reported metacognitive awareness (β > 0).

*H4*: Social interaction mediates the association between VR-enhanced collaborative learning and self-reported metacognitive awareness (indirect association > 0, 95% CI not containing zero).

*H5*: Learner engagement moderates the social interaction–metacognition association, such that the indirect association is stronger for more highly engaged learners.

## 3 Methodology

### 3.1 Research design and participants

This study employed a cross-sectional survey design with structural equation modeling (CB-SEM) for data analysis. Because the design was cross-sectional and non-experimental, all reported relationships should be interpreted as associational rather than causal. Participants were 520 Chinese university EFL students (M_age = 21.4, SD = 2.1; 57.3% female) enrolled in VR-enhanced collaborative English writing courses at three universities in mainland China. Participants had a minimum of one semester of VR-enhanced learning experience. G*Power analysis indicated that a sample of 520 was sufficient to detect medium effect sizes (*f*^2^ = 0.15) with 95% power at *α* = 0.05 in SEM analyses with five latent variables, consistent with current recommendations for SEM sample size (Wolf et al., 2013; Kyriazos, 2018).

#### 3.1.1 VR learning environment

The VR-enhanced collaborative writing courses were delivered using head-mounted displays (HMDs) operating in six-degrees-of-freedom mode. Two of the three participating institutions used Pico Neo 3 Pro Eye headsets; the third used HTC Vive Focus 3. All sites used a multi-user VR collaborative learning platform supporting synchronous voice chat, shared virtual whiteboards, document co-editing, and avatar-based peer interaction. Typical sessions involved 4–6 students per virtual classroom working on collaborative writing tasks such as joint argumentative essay planning, peer review of draft paragraphs, role-play discussions of source materials, and structured debate scenarios. Each VR session lasted approximately 45 min and was conducted twice per week across one academic semester (16 weeks), yielding approximately 24 h of cumulative VR-mediated learning per participant. Instructors joined VR sessions as facilitators, providing scaffolding, prompting metacognitive reflection, and monitoring task progress. Task design was guided by collaborative VR principles articulated by Paulsen et al. (2024), with particular emphasis on epistemic interdependence and synchronous co-construction. To standardize the learning environment across the three institutions, all sites ran the same version of the VR collaborative platform, followed an identical task script and instructor facilitation protocol, and configured both headset models (Pico Neo 3 Pro Eye and HTC Vive Focus 3) with equivalent six-degrees-of-freedom tracking, display, and audio settings; the two devices were confirmed comparable in a pre-study technical check.

#### 3.1.2 Sampling and recruitment

Participants were recruited from VR-enhanced collaborative writing courses at three Chinese universities representing different institutional types: one comprehensive research-intensive 985 Project university (*n* = 174, 33.5%), one teaching-oriented 211 Project university (*n* = 191, 36.7%), and one regional applied university (*n* = 155, 29.8%). Within each institution, the courses were offered as elective components of the regular English curriculum, and participation in the survey was voluntary, anonymous, and independent of course assessment. Of 561 returned questionnaires, 41 were removed prior to analysis owing to substantial missing data (>15% of items) or evidence of careless responding (e.g., failed attention checks or invariant response patterns), yielding a final analytic sample of *N* = 520. Item-level missingness in the retained sample was low (1.8% of all data cells; no individual item exceeded 3.6% missing), and missing values were handled using full-information maximum likelihood (FIML) estimation under the robust maximum likelihood (MLR) estimator, which yields unbiased parameter estimates under the missing-at-random assumption.

#### 3.1.3 Ethics

This study was conducted with human participants only; no animal subjects were involved. Participation was voluntary and anonymous, and participants were informed of the purpose of the study prior to completing the questionnaire.

### 3.2 Measures

All scales were administered in Chinese. Following Brislin’s translation procedure, two bilingual applied linguists independently forward-translated the English source instruments into Chinese, and a third bilingual translator back-translated the reconciled version into English; remaining discrepancies were resolved through discussion until semantic, idiomatic, and contextual equivalence was achieved. The Chinese version was then reviewed by a panel of three EFL experts for clarity and cultural appropriateness and piloted with 32 students comparable to the target sample, whose feedback was used to refine item wording. Pilot internal-consistency estimates were satisfactory for all four scales (Cronbach’s *α* > 0.80). A formal content validity index assessment was not conducted.

VR-Enhanced Collaborative Learning was assessed with a 12-item scale adapted from existing instruments on technology-mediated collaborative learning (Roe et al., 2025; Wiboolyasarin et al., 2024), measuring immersive presence, perceived collaborative affordances, and task authenticity (*α* = 0.88). Social Interaction Quality was measured with an 8-item scale assessing frequency and quality of peer interaction within VR environments, adapted from collaborative writing research (Peng et al., 2023; Mujtaba et al., 2021; α = 0.86). Self-reported metacognitive awareness was assessed with a 10-item scale adapted from the Self-reported metacognitive awareness Inventory, measuring metacognitive knowledge and regulation (Abdelaty et al., 2025; Zainuddin et al., 2026; α = 0.91). Learner Engagement was assessed with a 9-item scale measuring behavioral, cognitive, and emotional engagement dimensions (Koltovskaia, 2020; Wang et al., 2025; α = 0.87). All items used a 5-point Likert scale (1 = strongly disagree, 5 = strongly agree).

Operational distinction between the independent variable and the mediator. Because both constructs concern interaction, we separate them explicitly. VR-Enhanced Collaborative Learning indexes learners’ perceptions of the immersive environment’s affordances for collaboration (e.g., immersive presence, the shared virtual workspace, and task authenticity)—that is, the technological-pedagogical context. Social Interaction Quality indexes the perceived quality of the actual peer exchanges that took place within that context (e.g., responsiveness, negotiation of meaning, and joint co-construction)—that is, the interactional process the environment affords. The two are conceptually ordered as context → process rather than as interchangeable measures of “interaction.” Sample items: VR-ECL—e.g., “In the VR environment, I felt as if I were physically present in the same space as my classmates,” and “The shared virtual workspace allowed us to build on each other’s contributions”; SI—e.g., “My group members responded promptly to my ideas during the collaborative tasks,” and “We negotiated meaning when we disagreed about how to express something.” Full item sets for all four scales are provided in Appendix A. Discriminant validity between the two constructs is reported in Section 4.1 and Supplementary Table S2.

### 3.3 Analytical approach

CB-SEM was conducted using Mplus 8.8 with the MLR estimator to account for possible non-normality. Confirmatory factor analysis (CFA) was first conducted to establish measurement model fit prior to structural model testing, following established two-step modeling procedures. The indirect association of VR with self-reported metacognitive awareness through social interaction was estimated using 5,000-resample bootstrap confidence intervals. Model fit was evaluated using CFI, TLI, RMSEA, and SRMR, with thresholds of CFI/TLI > 0.95, RMSEA < 0.06, and SRMR < 0.08 considered indicative of acceptable fit (Lee, 2022; Zhang and Hyland, 2025). Because students were nested within intact classes (22 classes across the three institutions), all models were estimated with the TYPE = COMPLEX option in Mplus, with class membership specified as the clustering variable to obtain cluster-robust standard errors that adjust for the non-independence of observations within classes.

The moderated mediation hypothesis was tested using latent moderated structural equations (LMS; Klein and Moosbrugger, 2000) as implemented through the XWITH command in Mplus, with ALGORITHM = INTEGRATION (with 15 Gauss-Hermite integration points per latent dimension). LMS estimates the latent interaction term directly from the multivariate distribution of latent indicators without requiring product-indicator construction, which has been shown to produce less biased estimates and more accurate standard errors than product-indicator approaches under non-normal data (Cheung et al., 2021). Specifically, a latent interaction term (SI × LE) was specified using the syntax “INT | SI XWITH LE,” and the association of this interaction with self-reported metacognitive awareness was estimated to test the moderating role of learner engagement on the social interaction–metacognition pathway. The index of moderated mediation (Hayes, 2015) was computed as the product of the VR → SI path coefficient and the SI × LE → MC interaction coefficient, with bootstrap confidence intervals.

Common method bias was assessed through three complementary procedures to address recent concerns that Harman’s single-factor test alone is insufficient (Kock, 2015; Podsakoff et al., 2024; Williams et al., 2010). First, Harman’s single-factor test was conducted as a preliminary check. Second, a common latent factor (CLF) was specified in CFA, and standardized loadings, model fit, and a chi-square difference test were compared between models with and without the CLF, following Williams et al. (2010). Third, full collinearity variance inflation factors (VIFs) were computed for all constructs following Kock’s (2015) procedure, with VIFs below the conservative threshold of 3.3 indicating that common method variance is unlikely to bias structural estimates. Convergent validity was evaluated via average variance extracted (AVE > 0.50) and composite reliability (CR > 0.70). Discriminant validity was assessed through the heterotrait-monotrait ratio (HTMT < 0.85).

## 4 Results

### 4.1 Descriptive statistics, measurement model, and common method bias

Table 1 presents sample demographics. Table 2 presents means, standard deviations, and intercorrelations. CFA results confirmed acceptable model fit: χ^2^(218) = 486.7, χ^2^/df = 2.23, CFI = 0.972, TLI = 0.968, RMSEA = 0.049 (90% CI [0.043, 0.055]), SRMR = 0.052. All factor loadings were significant (*p* < 0.001) and ranged from 0.72 to 0.85. AVE values exceeded 0.50 for all constructs (range: 0.61–0.64), and CR values exceeded 0.70 (range: 0.93–0.95), supporting convergent validity. HTMT ratios ranged from 0.51 to 0.68, all below the conservative threshold of 0.85. Intraclass correlations for the study variables were small (ICC range = 0.04–0.09), and design effects were below 2.0 for all constructs (range = 1.21–1.78), indicating that the nesting of students within classes had a negligible impact on the standard errors; substantive conclusions were unchanged under the cluster-robust (TYPE = COMPLEX) specification. Full item-level standardized loadings together with construct-level AVE and CR are reported in Supplementary Table S1, and the complete heterotrait–monotrait (HTMT) matrix in Supplementary Table S2. The largest pairwise HTMT, between VR-enhanced collaborative learning and social interaction, was 0.68, remaining below the 0.85 criterion and thus supporting discriminant validity between the two interaction-related constructs.

**Table 1:** Demographic characteristics of the sample (*N* = 520).

Variable	Category	*n* (%)
Gender	Female	298 (57.3%)
	Male	218 (41.9%)
	Non-binary/Other	4 (0.8%)
Grade	Sophomore	168 (32.3%)
	Junior	204 (39.2%)
	Senior	148 (28.5%)
VR Experience	1–2 semesters	312 (60.0%)
	>2 semesters	208 (40.0%)
University type	985 Project (research-intensive)	174 (33.5%)
	211 Project (teaching-oriented)	191 (36.7%)
	Regional applied	155 (29.8%)

**Table 2:** Descriptive statistics and intercorrelations among study variables.

Variable	*M*	SD	1	2	3	4
1. VR collaborative learning	3.81	0.62	—			
2. Social interaction	3.74	0.58	0.52**	—		
3. Self-reported metacognitive awareness	3.69	0.64	0.48**	0.43**	—	
4. Learner engagement	3.77	0.61	0.41**	0.39**	0.46**	—

**p < 0.01 (two-tailed).

Three common method bias diagnostics yielded converging evidence that common method variance did not substantially bias the findings. First, Harman’s single-factor test indicated that a single factor accounted for 22.8% of the total variance, well below the 50% threshold of concern. Second, the common latent factor (CLF) approach indicated that adding a CLF to the measurement model produced only marginal changes in standardized loadings (Δλ range: 0.02–0.06; all below the 0.20 threshold of concern), with the CLF accounting for 14.3% of total item variance. The chi-square difference test between the CFA model with versus without the CLF was significant (Δχ^2^(22) = 38.6, *p* = 0.015), but the substantive loadings remained virtually unchanged and the CLF model retained acceptable fit (CFI = 0.974, RMSEA = 0.047), suggesting that any method variance present did not bias the substantive factor structure. Third, full collinearity VIFs for all constructs ranged from 1.42 to 2.18, all below Kock’s (2015) conservative threshold of 3.3. Taken together, these procedures indicate that common method variance is unlikely to materially affect the substantive conclusions.

### 4.2 Structural model and hypothesis testing

**Figure 1:**
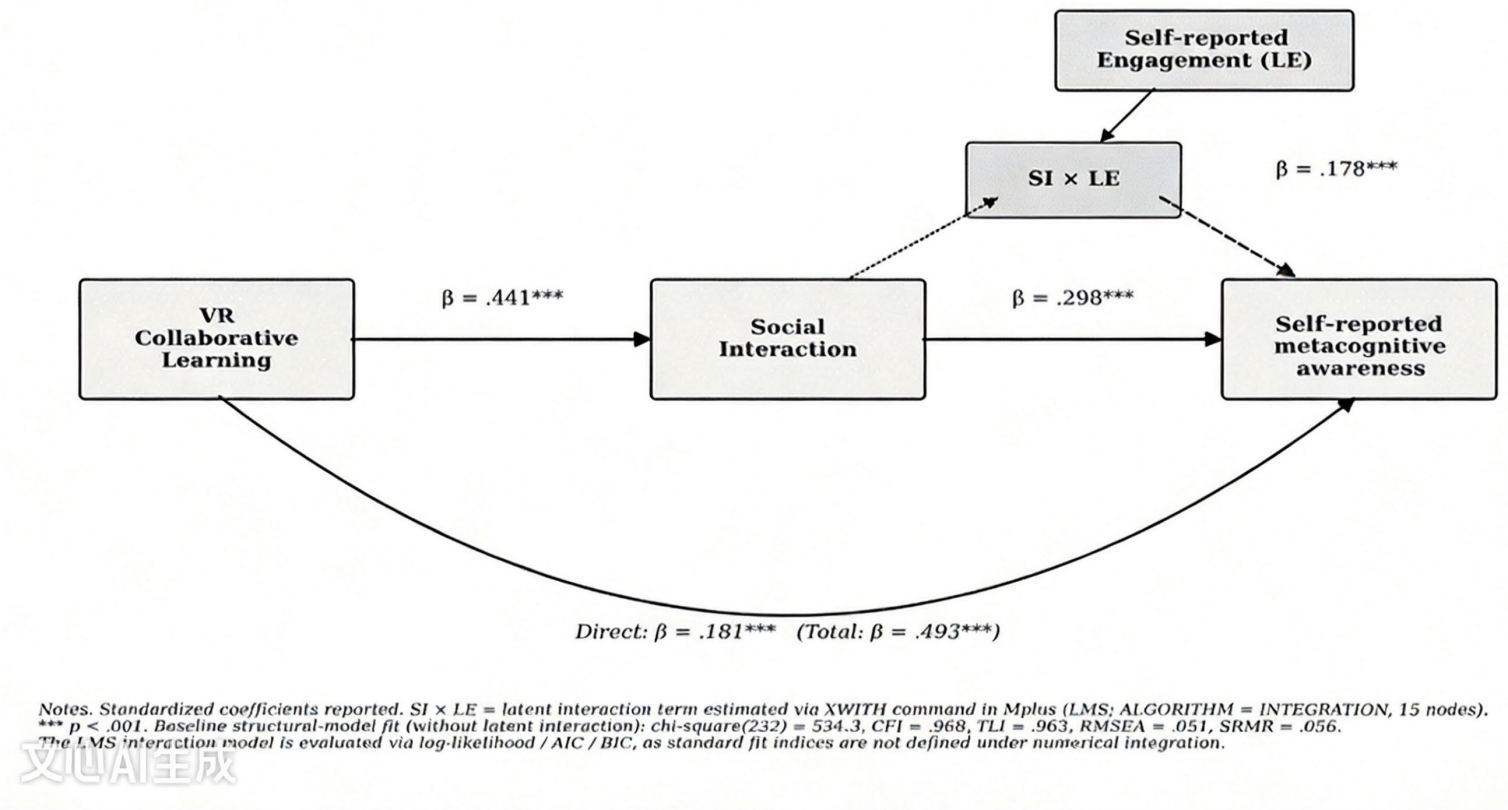
SEM path diagram with standardized path coefficients. VR = VR-enhanced collaborative learning; SI = Social Interaction; MC = Self-reported metacognitive awareness; LE = Learner Engagement; SI × LE = latent interaction term (XWITH in Mplus). Solid arrows = hypothesized paths; dashed arrow = moderating path. ***p < 0.001.

The structural model demonstrated good fit: χ^2^(232) = 534.3, χ^2^/df = 2.30, CFI = 0.968, TLI = 0.963, RMSEA = 0.051 (90% CI [0.045, 0.056]), SRMR = 0.056. Figure 1 presents the SEM path diagram with standardized path coefficients. Table 3 presents model fit indices. Table 4 presents path coefficients.

**Table 3:** Structural model fit indices.

Index	Value	Recommended threshold
χ^2^/df	2.30	<3.0
CFI	0.968	>0.95
TLI	0.963	>0.95
RMSEA	0.051	<0.06
SRMR	0.056	<0.08

Fit indices in this table are for the baseline structural model (without the latent interaction term). The latent moderated structural equations (LMS) model with the SI × LE interaction is evaluated via log-likelihood, AIC, and BIC, because standard fit indices are not defined under numerical integration. Comparison of the LMS model versus the baseline yielded ΔAIC = −18.4 and ΔBIC = −9.6 in favor of the LMS model.

**Table 4:** Structural path coefficients and latent interaction term.

Path	*β*	SE	*t*	*p*
VR → Metacognition (total)	0.312	0.048	6.50	<0.001
VR → Social Interaction	0.441	0.052	8.48	<0.001
Social Interaction → Metacognition	0.298	0.054	5.52	<0.001
SI × LE → Metacognition (XWITH)	0.178	0.038	4.68	<0.001
VR → Metacognition (direct)	0.181	0.043	4.21	<0.001

Standardized coefficients reported. SI × LE represents the latent interaction term estimated via the XWITH command in Mplus using latent moderated structural equations (LMS).

VR-enhanced collaborative learning was significantly and positively associated with self-reported metacognitive awareness (*β* = 0.312, SE = 0.048, *p* < 0.001), supporting H1. VR was also significantly associated with social interaction (*β* = 0.441, SE = 0.052, *p* < 0.001), supporting H2. Social interaction was significantly associated with self-reported metacognitive awareness (*β* = 0.298, SE = 0.054, *p* < 0.001), supporting H3. The direct association between VR and metacognition, after accounting for the mediation pathway, was *β* = 0.181 (SE = 0.043, *p* < 0.001), indicating partial mediation.

### 4.3 Mediation and moderated mediation

Bootstrap analysis (5,000 resamples) confirmed the indirect association of VR with self-reported metacognitive awareness through social interaction: indirect association = 0.131, 95% CI [0.078, 0.192], supporting H4. The exclusion of zero from the confidence interval indicates mediation. The conditional indirect association was significant at high engagement (+1 SD): *β* = 0.209, 95% CI [0.153, 0.268]; medium engagement (M): *β* = 0.131, 95% CI [0.078, 0.192]; and low engagement (−1 SD): *β* = 0.053, 95% CI [0.012, 0.098]. The latent interaction term (social interaction × learner engagement) was significantly associated with self-reported metacognitive awareness (*β* = 0.178, SE = 0.038, *p* < 0.001; Table 4). The index of moderated mediation was significant: IMM = 0.078, 95% CI [0.041, 0.122], supporting H5. The completely standardized indirect association was 0.131, and the proportion of the total association of VR with self-reported metacognitive awareness mediated by social interaction was 42.0% (0.131/0.312), indicating a substantial but partial mediation pattern.

**Figure 2:**
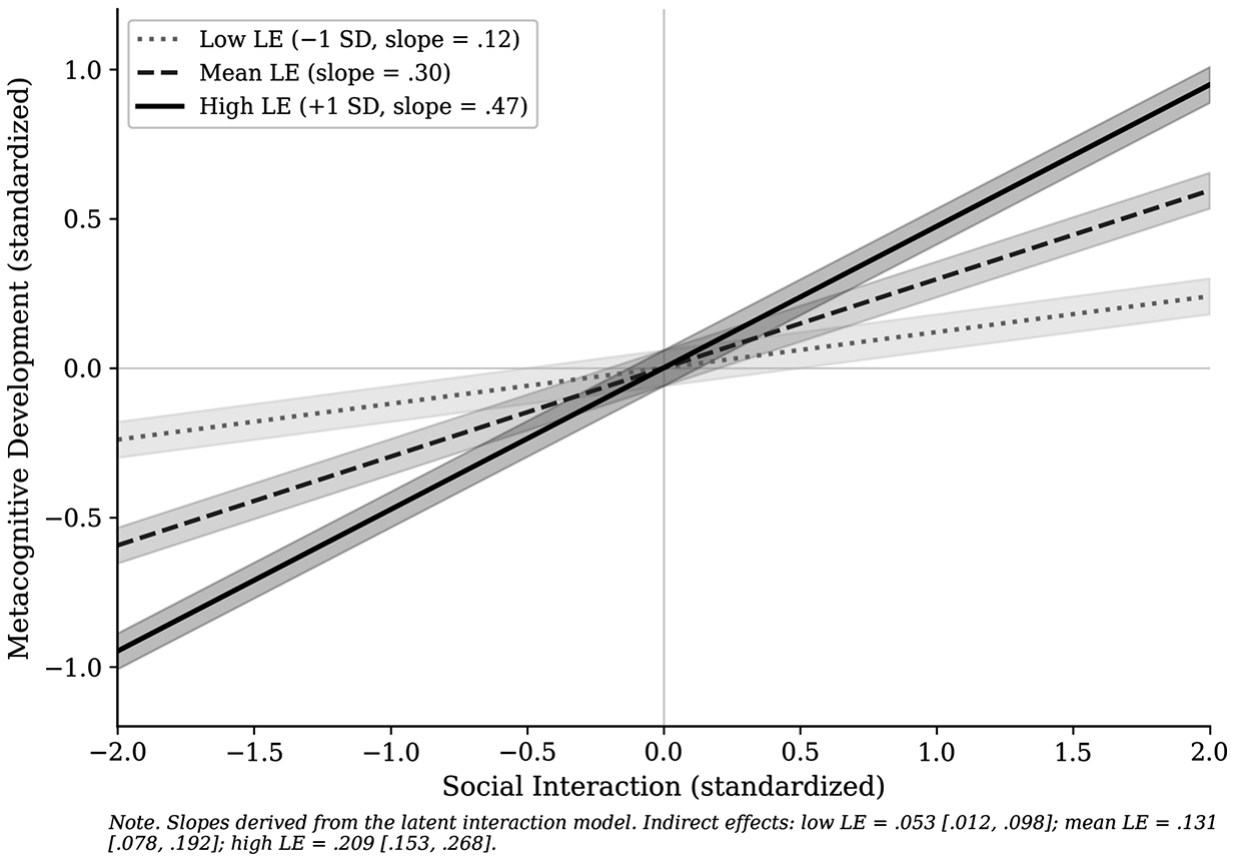
Simple slopes: conditional association of social interaction with Self-reported metacognitive awareness. Conditional association of social interaction with self-reported metacognitive awareness at low (−1 SD), mean, and high (+1 SD) levels of learner engagement. Slopes derived from the latent moderated structural equations model. Slopes for high engagement are significantly steeper than for low engagement.

Table 5 and Figure 2 summarize the moderated mediation associations. The Johnson-Neyman floodlight analysis indicated that the moderation was significant for learner engagement scores above 2.97 (on the 5-point scale), encompassing approximately 78.4% of the sample.

**Table 5:** Conditional indirect associations of VR with self-reported metacognitive awareness via social interaction.

Learner engagement level	Indirect association	SE	95% CI lower	95% CI upper
Low (−1 SD = 3.16)	0.053	0.022	0.012	0.098
Medium (*M* = 3.77)	0.131	0.029	0.078	0.192
High (+1 SD = 4.38)	0.209	0.030	0.153	0.268

Bootstrap 95% confidence intervals based on 5,000 resamples.

## 5 Discussion

### 5.1 VR-enhanced collaborative learning and self-reported metacognitive awareness

Consistent with theoretical accounts of VR as a cognitively enriching environment (Roe et al., 2025; Watts, 2025), VR-enhanced collaborative learning was positively associated with self-reported metacognitive awareness (H1). The immersive, task-authentic character of these environments may foster the productive epistemic uncertainty Watts (2025) termed cognitive debt—a state that can accompany metacognitive engagement—and the pattern resonates with work linking experiential, immersive technologies to metacognitive reflection (Zainuddin et al., 2026). Because the design was cross-sectional, however, this association cannot be read as evidence that VR causally produces self-reported metacognitive awareness; the temporal ordering of the constructs is not identifiable from these data.

### 5.2 Social interaction as a mediator

Social interaction emerged as a significant mediator (H4), aligning with sociocultural predictions that higher cognitive functions are socially constructed before being internalized (Mujtaba et al., 2021; Peng et al., 2023). Substantively, this implies that VR relates to self-reported metacognitive awareness less through solitary engagement with the virtual environment than through the qualitatively richer peer exchange it affords, which in turn supports metacognitive internalization. The pattern extends Peng et al.’s (2023) finding that collaborative feedback processing engages learners more deeply than individual processing, and Mujtaba et al.’s (2021) account of how the social negotiation of linguistic norms consolidates accuracy.

The structural pathways underlying this mediation further support H2 (VR → social interaction, *β* = 0.441, *p* < 0.001) and H3 (social interaction → self-reported metacognitive awareness, *β* = 0.298, *p* < 0.001), which together constitute the proposed mechanistic chain. The partial mediation pattern (direct association *β* = 0.181, *p* < 0.001; indirect association *β* = 0.131) suggests that social interaction is one, but not the only, pathway through which VR-enhanced collaborative learning may relate to metacognitive outcomes. Other plausible mechanisms include the metacognitive demands of navigating three-dimensional virtual environments, the heightened attention and presence associated with VR immersion, and the multimodal richness of VR stimuli (Roe et al., 2025). Sang and Zou (2023) similarly found that collaborative joint production was associated with language learning gains beyond what a purely socially mediated pathway could explain.

### 5.3 The moderating role of learner engagement

The finding that learner engagement moderated the social interaction–metacognition pathway (*β* = 0.178, *p* < 0.001) supports H5 and extends previous research on engagement as a boundary condition for technology-enhanced learning outcomes (Koltovskaia, 2020; Wang et al., 2025; Zhang and Hyland, 2018). The Johnson-Neyman analysis, indicating that moderation was significant for learner engagement scores above 2.97 (encompassing 78.4% of the sample), suggests that the moderated mediation association was broadly operative across the sample rather than confined to an extreme subgroup. This finding complements Zhang’s (2020) documentation of engagement as a determinant of the productive use of automated feedback systems and Zhang and Hyland’s (2025) demonstration that digital literacy shapes the quality of learner processing of AWE feedback.

The stronger indirect association among more highly engaged learners (*β* = 0.209 vs. *β* = 0.053 for low engagement) is theoretically interpretable as suggesting that engagement amplifies learners’ use of VR’s social interactional affordances. More highly engaged learners may participate more actively in collaborative tasks, generate more substantive peer dialogue, and more deeply internalize metacognitive insights that emerge through social negotiation. This interpretation is consistent with Tsao’s (2021) finding that writing self-efficacy, a construct closely related to behavioral engagement, predicted the quality of engagement with written corrective feedback, and with Wang et al.'s (2025) documentation that motivational engagement was the strongest predictor of L2 writing outcomes.

### 5.4 Implications

#### 5.4.1 Theoretical contributions

Theoretically, this study contributes a moderated mediation framework for understanding how VR-enhanced collaborative learning environments may relate to self-reported metacognitive awareness. The identification of social interaction as a mediator and learner engagement as a moderator advances beyond binary assessments of VR effectiveness to specify the conditions under which VR’s associations with metacognitive outcomes are most pronounced. The framework refines sociocultural accounts of technology-enhanced learning by specifying social interaction quality, rather than mere exposure to a technology, as the proximal mechanism through which VR affordances appear to translate into metacognitive gains. It also extends boundary-condition theorizing on engagement by demonstrating that engagement operates not only as an antecedent of learning outcomes but as a moderator of the social-mediation pathway itself. These contributions speak to broader theoretical conversations on the cognitive and social mechanisms of technology-enhanced learning (Lee et al., 2025; Watts, 2025).

#### 5.4.2 Pedagogical implications

Pedagogically, the findings suggest that VR implementations should prioritize collaborative task designs that maximize social interaction quality, rather than focusing exclusively on immersive individual experiences. Instructors should scaffold learner engagement through pre-VR orientation activities, reflective debriefs, and formative feedback that activates metacognitive monitoring during VR tasks. Zainuddin et al. (2026) demonstrated that e-portfolio assessment was associated with metacognitive self-regulation among pre-service teachers; integrating portfolio-based reflection into VR courses may further strengthen the social interaction–metacognition pathway documented here. Because the moderated indirect association is weakest for low-engagement learners (*β* = 0.053), low-engagement students may require supplementary motivational and social scaffolding strategies, including pre-VR goal-setting protocols, peer accountability structures, and formative engagement monitoring during VR sessions, to benefit equitably from VR-enhanced collaborative interaction.

#### 5.4.3 Technological design implications

From a design perspective, the present findings support three evidence-informed recommendations. These design recommendations are derived from associational, cross-sectional evidence and should be regarded as hypotheses to be empirically validated in experimental or longitudinal designs before being treated as causal prescriptions. First, collaborative VR task design should prioritize genuine epistemic interdependence, in which no individual can complete the task without substantive information exchange with peers (Paulsen et al., 2024; Van der Meer et al., 2023). Task designs that require joint problem formulation, collaborative hypothesis testing, and shared knowledge construction are most likely to generate the quality of social interaction that, as the present findings indicate, is associated with self-reported metacognitive awareness. Merely placing learners in a shared virtual environment without structuring collaborative interdependence is unlikely to produce the social interaction quality necessary to activate this metacognitive pathway. Second, metacognitive scaffolding tools, including learning analytics dashboards, metacognitive strategy prompts, and structured reflection protocols, should be integrated directly into VR learning environments rather than deployed as post-hoc supplements (Chen, 2025; Geng and Yamada, 2025). In VR contexts, the unique affordance of immersive presence may amplify the effects of these tools by sustaining attentional engagement with metacognitive reflection prompts. Third, VR-based collaborative learning platforms should incorporate adaptive engagement-monitoring features, given the substantial differential documented here between high- and low-engagement learners. Engagement-differentiated design, combining VR immersion with targeted engagement-building interventions calibrated to learner profiles, offers a promising direction for both future research and platform development in technology-enhanced language learning.

## 6 Conclusion

This study examined the associations among VR-enhanced collaborative learning, social interaction, learner engagement, and self-reported metacognitive awareness in a sample of 520 Chinese university EFL students. CB-SEM analyses with latent moderated structural equations confirmed that social interaction mediates the VR–metacognition association, and that learner engagement moderates this mediation, such that the indirect association is strongest for more highly engaged learners. These findings contribute to sociocultural accounts of technology-enhanced self-reported metacognitive awareness and provide practical guidance for designing VR-based collaborative learning environments. As the design was cross-sectional, the relationships reported are associational and do not warrant causal interpretation.

Several limitations merit acknowledgment. First, the cross-sectional design precludes causal inference; longitudinal designs are needed to map the temporal unfolding of VR’s metacognitive associations. Second, the sample, drawn from Chinese university EFL contexts, limits generalizability; the documented moderating role of cultural-linguistic congruence (Hoang, 2025) suggests that VR-related associations may differ across cultural settings. Third, self-report measures of metacognition may be subject to social desirability and recall biases; future research should complement self-report with behavioral or process-tracing measures of metacognitive activity, such as think-aloud protocols, log-file analyses, or eye-tracking. Fourth, while three complementary common method bias diagnostics converged in suggesting that method variance did not materially bias the substantive findings, the use of a single source and single occasion remains a structural limitation that future multi-source or multi-wave designs should address. Fifth, the use of two different head-mounted display models across institutions, together with heterogeneity among the three universities, may have introduced unmodeled variability; although a common platform and task protocol were used, future studies should standardize or experimentally control hardware and institutional context. Notwithstanding these limitations, the present study provides an empirically grounded moderated mediation framework for ongoing inquiry into immersive collaborative learning.

## Data Availability

The original contributions presented in the study are included in the article/supplementary material, further inquiries can be directed to the corresponding author/s.
